# Society Meetings

**Published:** 1911-10

**Authors:** 


### SOCIETY MEETINGS

---

### Mississippi Valley Medical Association

Plans are being perfected for the big medical meeting in Nashville, October 17, 18, 19, 1911. The local arrangements are in the hands of Dr. John A. Witherspoon and an able committee.

This Association was originally the Tri-State Medical Society of Kentucky, Indiana, and Tennessee, but the scope of its activities was later enlarged, and while its name is of the Valley, it embraces in its membership men from the Atlantic to the Rockies.

Dr. Henry Enos Tuley, of Louisville, Ky., has been Secretary of the Association since the meeting held in Louisville in 1897, and Dr. S. C. Stanton, of Chicago, has served continuously as Treasurer since 1902.

Among the Southern doctors on the program for the Nashville meeting are the following: Louisville—Drs. Louis Frank, Walter F. Boggess, W. D. Haines, E. T. Bruce, Thomas Hunt Stucky; August, Ga.—Dr. Thomas D. Coleman; Asheville, N. C. —Drs. Charles L. Minor, Silvio von Ruck; St. Louis—Drs. George Dock, William Engelbach, Brandsford Lewis; Memphis, Tenn.—Drs. Frank Jones, Frank D. Smythe; New Orleans—Dr. E. M. Hummel; Richmond, Va.—Dr. Robert C. Bryan; Cincinnati, O.—Drs. Earl Harlan, E. O. Smith; Lexington, Ky.—Dr. J. A. Stucky. The following Chicago men are on the program: Drs. Robert H. Babcock, Alexander C. Wiener, Arthur Elliott, Channing W. Barrett, William Thompson, Fenton Benedict Turck, Bayard Holmes, Robert B. Preble, Cary Culbertson.

---

Tuesday, October 17. Quarterly meeting of Monroe County Medical Society. Program not ready. Largely a business meeting. Dr. W. B. Jones, presiding and Dr. A. C. Snell, secretary. Held in evening at Hotel Seneca.

Wednesday, October 18. Central New York Medical Society. All day session. Hotel Seneca. Dr. W. T. Mulligan presiding. Papers by Dr. G. W. Crile, Cleveland; Dr. J. B. Murphy, Chicago; Dr. Roswell Park, Buffalo; Dr. W. S. Baer, Baltimore and others not definitely determined.

Thursday, October 19. Seventh District Branch of the Medical Society of the State of New York. Annual meeting; all day session. Hotel Seneca.

*Preliminary Program.*

1. "Tuberculosis of the Pelves in Women," Earl P. Lothrop, M.D., Buffalo.

2. "Surgical Tuberculosis," James A. MacLeod, M.D., Buffalo.

3. "General Considerations in Tubercular Treatment," Norman K. MacLeod, Buffalo.

4. "Salvarsan vs. Mercury," E. Wood Ruggles, M.D., Rochester.

5. "Sanitation of Army Camps," Charles O. Boswell, M.D., Rochester.

6. "*X*-Ray Examinations as an Aid in Diagnosis," M. B. Palmer, M.D., Rochester.

7. "Influenzal Arthritis," J. P. Creveling, M.D., Auburn.

8. "Proctoclysis and an Apparatus that Works Satisfactorily," H. J. Knickerbocker, M.D., Geneva.

9. "Diagnosis of Diseases about the Waist Line," J. R. Culkin, M.D., Rochester.

10. "A Report of a Case of Paraplegia Inferior, with Treatment and Result," E. C. Foster, Penn Yan.

11. Subject to be announced, Nathan Jacobson, M.D., Syracuse.

12. Subject to be announced, William W. Skinner, M.D., Geneva.

Social. On both days a luncheon will be served by the local society to both visiting organizations and a dinner (subscription) on the evening between the two meetings.

---

THE next meeting of the Southern Surgical and Gynecological Association will be held in Washington City, December 12, 13 and 14th.

---

**Buffalo Academy of Medicine—Meeting for October, 1911**

October 3.—Surgical Section: Some Conditions Simulating Disease of the Spine or Hip; Dr. Prescott Le Breton. Use of Intraarticular Silk Ligaments for Limiting Movements in the Paralytic Joint; Dr. Bernard Bartow. Joint Fractures—Lantern Slide Demonstration; Dr. William Ward Plummer.

October 10.—Medical Section: The Thyroid Gland and the Relation of Basedow's Disease to Surgery: Dr. C. F. Hoover, Cleveland.

October 17.—Section of Obstetrics and Gynecology: Observation of the Pelvic Floor; Dr. Irving W. Potter. Indications for and Technic of Artificial Sterilization of Women; Dr. T. H. McKee.

October 24.—Pathological Section, Kidney and Bladder Stone, Dr. Hugh H. Young, Baltimore.

